# Ab Initio Study of Structural, Electronic and Magnetic Properties of TM&(B@C_60_) (TM = V, Cr) Sandwich Clusters and Infinite Molecular Wires

**DOI:** 10.3390/nano12162770

**Published:** 2022-08-12

**Authors:** Jie Ji, Tianxia Guo, Liyan Qian, Xiaokang Xu, Huanning Yang, Yue Xie, Maoshuai He, Xiaojing Yao, Xiuyun Zhang, Yongjun Liu

**Affiliations:** 1 College of Physics Science and Technology, Yangzhou University, Yangzhou 225002, China; 2 College of Chemistry and Molecular Engineering, Qingdao University of Science and Technology, Qingdao 266042, China; 3 College of Physics and Hebei Advanced Thin Film Laboratory, Hebei Normal University, Shijiazhuang 050024, China

**Keywords:** sandwich complexes, magnetic, density functional theory

## Abstract

The geometrical structure, electronic and magnetic properties of B-endoped C_60_ (B@C_60_) ligand sandwich clusters, TM&(B@C_60_)_2_ (TM = V, Cr), and their one-dimensional (1D) infinite molecular wires, [TM&(B@C_60_)]_∞_, have been systematically studied using first-principles calculations. The calculations showed that the TM atoms can bond strongly to the pentagonal (η^5^-coordinated) or hexagonal rings (η^6^-coordinated) of the endoped C_60_ ligands, with binding energies ranging from 1.90 to 3.81 eV. Compared to the configurations with contrast-bonding characters, the η^6^- and η^5^-coordinated bonding is energetically more favorable for V-(B@C_60_) and Cr-(B@C_60_) complexes, respectively. Interestingly, 1D infinite molecular wire [V&(B@C_60_)-η^6^]_∞_ is an antiferromagnetic half-metal, and 1D [Cr&(B@C_60_)-η^5^]_∞_ molecular wire is a ferromagnetic metal. The tunable electronic and magnetic properties of 1D [TM&(B@C_60_)]_∞_ SMWs are found under compressive and tensile stains. These findings provide additional possibilities for the application of C_60_-based sandwich compounds in electronic and spintronic devices.

## 1. Introduction

Since the discovery of C_60_ in 1985 [1,2], various fullerenes and their derivatives have attracted great attention due to their extraordinary stability and unique chemical and physical properties [3,4,5,6,7]. Particularly, versatile polygons, such as pentagons and hexagons, in fullerenes enabled them to be potential ligands bonding in external metal elements. Using a laser vaporization method, Nakajima et al. [8,9,10] successfully synthesized TM-C_60_ (TM= 3*d* transition metal) complexes in the 1990s, and predicted that the TM*_n_*(C_60_)*_m_* clusters exhibit sandwich-like structures for *m* = *n* + 1, *n* ≤ 3 or ring-like structures for *m* = *n*, *n* > 3. These structure characteristics were later confirmed via theoretical studies [11]. In addition, the TM-C_60_ coordinated bonds in TM*_n_*(C_60_)*_m_* complexes were found to be dependent on the choice of TM atom [12,13]. However, differently from the comprehensively studied benzene (Bz)-ligand [10,14] or cyclopentadienyl (Cp)-ligand [15] sandwich complexes, most C_60_ sandwich complexes were confirmed to be non-magnetic or weakly magnetic [11,12,13], severely limiting their application in spintronic devices. Therefore, tuning the electronic and magnetic properties of fullerene-ligand sandwich complexes remains challenging. 

Different from organic planar ligands (C*_n_*H*_n_*, *n* = 5–8) [16,17,18,19,20,21,22], the cage configuration of fullerenes enable their large spaces to accommodate other atoms or molecules for forming various core/shell complexes [23,24,25,26,27,28,29,30,31,32], which endows novel electronic properties on them and their TM-fullerene sandwich derivatives. For example, 1D infinite molecular wires, [TM&(TM@C_60_)]_∞_ (TM = Ti-Ni), composed of metallofullerene (TM@C_60_)[23,33,34] and 3*d* TM atoms, were identified as displaying robust antiferromagnetic (AFM) semiconducting properties [20]. Unfortunately, the physical and chemical properties of sandwich isomers with different endoped fullerenes are rarely investigated. Herein, we explore the structure, electronic and magnetic properties of TM(B@C_60_)_2_ (TM = V, Cr) sandwich clusters, as well as their 1D molecular wires (SMWs), [TM&(B@C_60_)]_∞_, constructed by the fabricated core/shell structure, B@C_60_ [35], as a building block. All the V-(B@C_60_) and Cr-(B@C_60_) sandwich complexes with η^6^- or η^5^- are thermodynamically stable, with high binding energies. Among them, the η^6^- and η^5^-coordinated bonding configurations are the stable ones, respectively, for the V and Cr atom. Moreover, 1D [V&(B@C_60_)-η^6^]_∞_ SMW is an antiferromagnetic half-metal, and 1D [Cr&(B@C_60_)-η^5^]_∞_ molecular wire is a ferromagnetic metal.

## 2. Models and Method 

All the calculations were performed in the Vienna ab initio simulation package (VASP) [36,37] under the spin-polarized DFT framework. The exchange correlation interaction was described by the Perdew–Burke–Ernzerhof (PBE) [38] functional, and the interaction between valence electrons and ion nuclei was described by the projector–augmented wave potential (PAW) [39] method. In the process of calculations, the van der Waals (vdW) interaction was considered by using the DFT-D2 [40] method. In order to further consider the Coulomb interaction and exchange interactions of the *d*-electron in the transition metal atom, we adopted the GGA + U method [41], in which the parameter U was set to 3.0 Ev [42,43]. In order to find the magnetic ground state of the 1D [TM&(B@C_60_)]_∞_ SMWs, a 1 × 1 × 2 supercell consisting of two TM atoms and two (B@C_60_) units was used. The criteria for energy and atom force convergence were set to 10^−4^ eV and 0.01 eV/Å, respectively. To determine the magnetic ground states of the TM&(B@C_60_) clusters and molecular wires, diverse magnetic states with different magnetic moments were calculated and compared.

## 3. Results and Discussion

### 3.1. TM&(B@C_60_)_2_ (TM = V, Cr) Sandwich Clusters 

**Figure 1:**
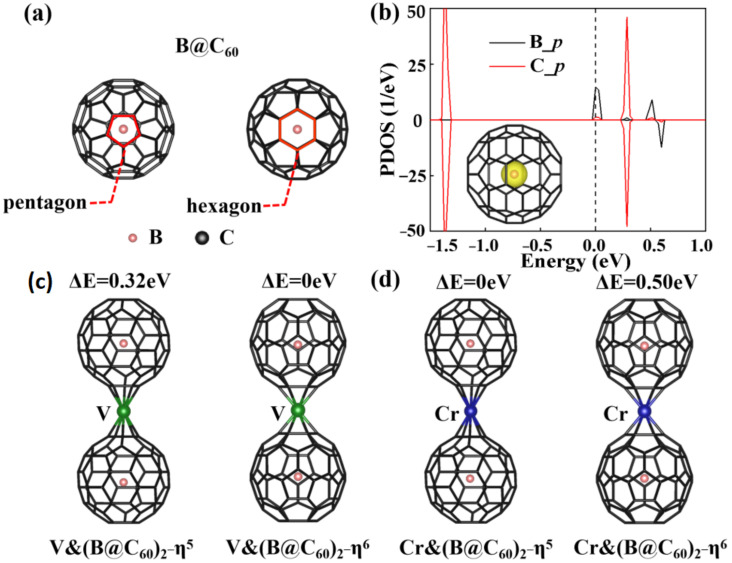
(**a**) Different views of B@C_60_; (**b**) the PDOS and spin density plot of B@C_60_ molecule; optimized structures of V&(B@C60)_2_ (**c**) and Cr&(B@C60)_2_ (**d**) with η5 and η6 bonding. ΔE is the energy difference between different bonding structures.

First, we explored the structural characters of the endohedral B@C_60_ cluster (see Figure 1a). Similar to the C_60_ molecule, the point group symmetry of the B@C_60_ molecule is Ih, with the B atom sitting on the mass center of C_60_. The diameter of B@C_60_ is 7.09 Å and the C-C bond length is 1.45 Å. As shown in the spin density plot (see Figure 1b), the B atom in B@C_60_ is spin-polarized with a local magnetic moment of 1.0 μ_B_. The partial density of state (PDOS) of B@C_60_ (Figure 1b) shows that the *p* states of the B atom is spin-polarized in the energy around the Fermi level, accounting for the 1.0 μ_B_ net magnetic moment. Two types of TM&(B@C_60_)_2_ configurations were considered: (i) TM&(B@C_60_)_2_-η^5^, in which the sandwiched TM atoms are bonded to two pentagonal rings of two B@C_60_ molecules forming η^5^-coordinate bonds; and (ii) TM&(B@C_60_)_2_-η^6^, in which the TM atoms are bonded to two hexagonal rings of two B@C_60_s forming η^6^-coordinate bonds. Figure 1c,d show the optimized structures of V&(B@C_60_)_2_ and Cr&(B@C_60_)_2_. Clearly, all the TM&(B@C_60_)_2_s favor normal sandwich configurations, with the TM atom sitting above the mass center of the pentagon or hexagon rings. For V&(B@C_60_)_2_, the η^6^ coordinated configuration is more stable than the η^5^ coordinated one, with an energy difference of 0.32 eV. On the contrary, the η^5^ coordinated configuration is energetically more stable for Cr&(B@C_60_)_2_, with approximately 0.50 eV less energy. 

**Figure 2:**
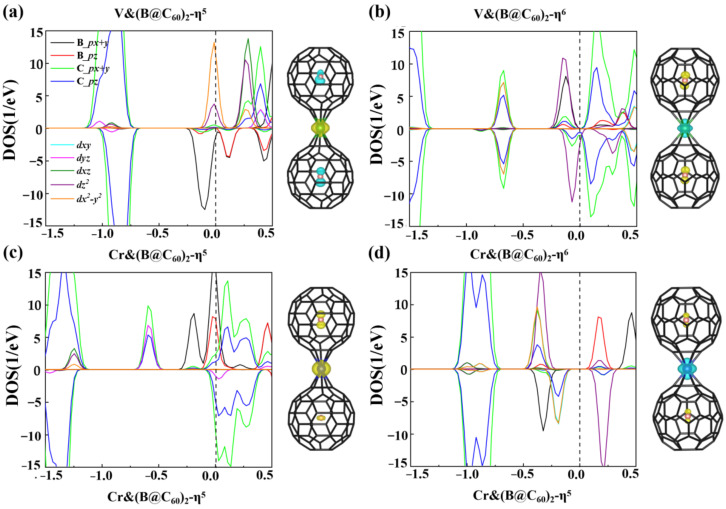
The spin density and PDOS of V&(B@C_60_)_2_ (**a**,**b**) and Cr&(B@C_60_)_2_ (**c**,**d**).

For V&(B@C_60_)_2_ and Cr&(B@C_60_)_2_, the distances of TM atoms from the mass center of the faced C*_n_* ring (*n* = 5, 6) (*d*_TM-C60_) to the nearest carbon rings are in the range of 1.73–2.00 Å (see Table 1), which are a bit larger than those in the TM-Bz (1.70 Å) [44], TM-Cp (1.72 Å–1.81 Å) [15] and TM-C_60_ (1.75 Å) [6] sandwich compounds. In the compounds, the *d*_TM-C60_s in η^5^ coordinated systems are longer than those in the η^6^ coordinated ones by around 0.21~0.28 Å. Moreover, the B atoms in the TM&(B@C_60_)_2_s (Figure 1c,d) deviate from the center of C_60_ with 0.02~0.08 Å (see Table 1). In order to investigate the stability of these TM&(B@C_60_)_2_ sandwich clusters, the binding energies (E_b_) are calculated based on the following formula:

*E_b_* =* E_TM&(B@C60)2_* − [*E_TM_ + 2E_B@C60_*] (1)

where E_TM_, E_B@C60_ and E_TM&(B@C60)2_ are the energies of the isolated TM atom, B@C_60_ molecule and TM&(B@C_60_)_2_, respectively. Shown in Table 1, the E_b_s of V&(B@C_60_)_2_ and Cr&(B@C_60_)_2_ with η^5^/η^6^ coordinated bonding are approximately −1.90/−2.23 eV and −3.81/−3.31 eV, respectively, implying that these sandwich clusters are energetically stable. Figure 2 plots the PDOS of the TM&(B@C_60_)_2_ (TM = Ti, V) clusters to explore the physical origin of their stability. For V&(B@C_60_)_2_-η^6^, strong C-*p* and V-*d_x2−y2_* orbitals hybridization are observed in the energy window of [−0.75, −0.60 eV], and the hybridization between B-*p* and V-*d_z2_* states are in the energy window of [−0.25, 0 eV] (see Figure 2b) below the Fermi level. While in the case of V&(B@C_60_)_2_-η^5^, relatively weaker B-*p* and V-*d_z2_* hybridization is found (see Figure 2a), which is responsible for its low stability. In Cr&(B@C_60_)_2_-η^6^, the hybridization between C-*p* orbitals and Cr-*d_x2-y2_*, *d_z2_* orbitals is observed in the energy of [−0.5, −0.1 eV]. In contrast, for Cr&(B@C_60_)_2_-η^5^, stronger *d*-*p* hybridization is found deep in the energy window below the Fermi level, around [−1.3, −1.2 eV] and [−0.6, −0.4 eV]. As a result, the most energetically stable configuration is Cr&(B@C_60_)_2_-η^5^.

To determine the magnetic ground states of these TM&(B@C_60_)_2_ clusters, we considered different spin states for each system (see Table S1 in the supporting information, SI). For V&(B@C_60_)_2_, the magnetic moment of its ground state is 3.0 μB and 1.0 μB in their η^5^/η^6^ coordinated configurations. Their second lower-energy isomers are found to have magnetic moments of 5.0 μB and 3.0 μB, which are less stable than the ground states by approximately 0.01 eV and are 0.03 eV higher in energy, respectively. In addition, for Cr&(B@C_60_)_2_-η^5^ and Cr&(B@C_6_0)_2_-η^6^, the magnetic moment of 6.0 μB and 2.0 μB is observed for their ground states, which are approximately 0.03 eV and 0.17 eV lower in energy, respectively, than their second higher-energy isomers with the same magnetic moment of 4.0 μB. The inset in Figure 2 shows the spin densities of V&(B@C_60_)_2_ and Cr&(B@C_60_)_2_. Clearly, the magnetic moments of both systems are mainly contributed to the B atoms and TM atoms. The B atom and TM atom for V&(B@C_60_)_2_-η^5^ and Cr&(B@C_60_)_2_-η^6^ contribute to opposite spins. In contrast, the same spins are found for the B atom and Cr atom in Cr&(B@C_60_)_2_-η^5^. As for V&(B@C_60_)_2_-η^6^, its magnetic moments mainly arise from two B atoms with opposite spins.

### 3.2. D infinite [TM&(B@C_60_)]_∞_ (TM = V, Cr) SMWs 

**Figure 3:**
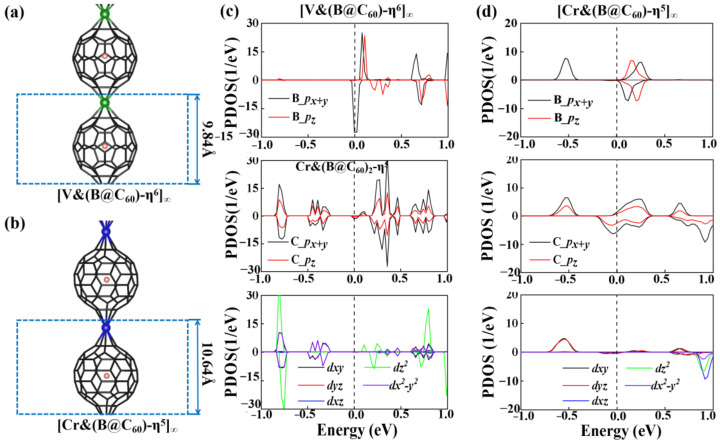
The optimized structures (**a**,**b**) and PDOS (**c**,**d**) of 1D [V&(B@C_60_)-η^6^]_∞_ and [Cr&(B@C_60_)-η^5^]_∞_ SMWs.

Figure 3a,b show the optimized structures of 1D [V&(B@C_60_)]_∞_ and [Cr&(B@C_60_)]_∞_. Here, respective 1D [V&(B@C_60_)]_∞_ and [Cr&(B@C_60_)]_∞_ with η^6^- and η^5^-coordinated configurations are considered. Similar to the TM&(B@C_60_)_2_ clusters, both 1D [TM&(B@C_60_)]_∞_ SMWs have normal sandwich structures. The lattice constants of 1D [V&(B@C_60_)-η^6^]_∞_ and [Cr&(B@C_60_)-η^5^]_∞_ SMWs are 9.84 Å and 10.64 Å, respectively (see Table 1 and Figure 3a,b). Meanwhile, B atoms in the endoped C_60_ are separate from the mass center of C_60_, with the deviation values (Δ*h*) of 0.32 Å and 0.09 Å, respectively. Table 1 shows that the d_TM-C60_ in 1D [V&(B@C_60_)-η^6^]_∞_ SMW and [Cr&(B@C_60_)-η^5^]_∞_ SMW are approximately 1.70 Å and 2.01 Å, respectively, and are a bit shorter than that in the finite sandwich clusters. 

**Figure 4:**
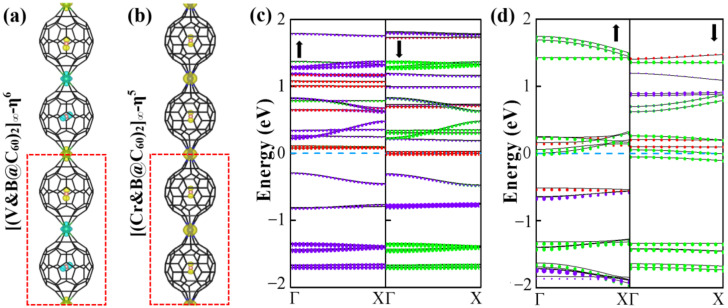
Spin densities (**a**,**b**) and decomposed band structures (**c**,**d**) of 1D [V&(B@C_60_)-η^6^]_∞_ and (**b**) [Cr&(B@C_60_)-η^5^]_∞_ SMWs, yellow and blue colors indicate up and down spins, respectively. red, green and violet colors represent B-*p*, C-*p* and TM-*d* orbitals, respectively, the size of the color balls is proportion to the contributions from the states.

To evaluate the stability of these SMWs, we defined the binding energy (E_b_) of SMWs as:

(2) $$E_{b}=E_{[}{{}_{TM\&(B@C60)]}}_{\infty }-E_{TM}-E_{B@C60}$$

where E_[TM&(B@C60)]∞_, E_TM_ and E_B@C60_ are the energies of [TM&(B@C_60_)]_∞_ SMWs, 3*d* TM atoms and B@C_60_ ligand, respectively. As shown in Table 1, the binding energies of [V&(B@C_60_)-η^6^]_∞_ and [Cr&(B@C_60_)-η^5^]_∞_ are about −5.24 eV and −8.67 eV, respectively, larger than that of the reported 1D organometallic and non-organometallic SMWs [15,22,45,46,47]. The Bader charge calculations indicate that their high stability is correlated with charge transfer from the TM atom to B@C_60_ molecule, which is about 1.22e and 1.09 e for V and Cr, respectively. Figure 3c,d present the PDOS of 1D [V&(B@C_60_)-η^6^]_∞_ SMW and [Cr&(B@C_60_)-η^5^]_∞_ SMW. Strong hybridization between C-*p* and V-*d_z2_*, *d_x2-y2_* orbitals are found in 1D [V&(B@C_60_)-η^6^]_∞_ SMW. As shown in Figure 4d, C-*p* and Cr-d*yz* of [Cr&(B@C_60_)-η^5^]_∞_ SMW are strongly hybridized.

**Figure 5:**
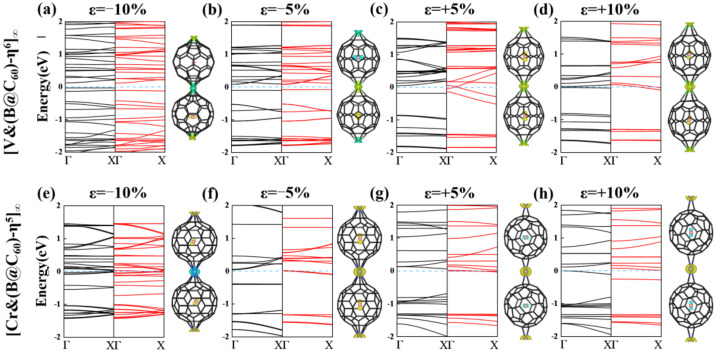
Band structures and spin density of 1D [V&(B@C_60_)-η^6^]_∞_ and (**b**) [Cr&(B@C_60_)-η^5^]_∞_ SMWs under several strains of −10% (**a**,**e**), −5% (**b**,**f**), +5% (**c**,**g**) and +10% (**d**,**h**); yellow and blue colors indicate up and down spins, respectively. The blue, black and red lines represent the Fermi level, the spin up and spin down electronic bands, respectively.

Furthermore, to determine the magnetic ground state of 1D [V&(B@C_60_)-η^6^]_∞_ and [Cr&(B@C_60_)-η^5^]_∞_ SMWs, a double cell was constructed to explore their FM and AFM configurations (see Figure 4a,b). Obviously, 1D [V&(B@C_60_)-η^6^]_∞_ SMW favors an AFM ground state, in which two nearby V-B atoms (V-B dimer) FM couple with each other, while AFM couples with its nearby dimer (see Figure 4a). The FM state is less stable by approximately 0.35 eV higher in energy. On the contrary, 1D [Cr&(B@C_60_)-η^5^]_∞_ SMW has a FM ground state, which is more stable than the AFM state by approximately 0.10 eV lower in energy (see Table S2). Moreover, 1D [V&(B@C_60_)-η^6^]_∞_ SWM is found to be an AFM half-metal, in which the spin-up and spin-down electronic states are semiconducting and conducting, respectively (see Figure 4c), while 1D [Cr&(B@C_60_)-η^5^]_∞_ SMW is a FM metal(see Figure 4d). Finally, we explored the electronic and magnetic properties of the most stable 1D [TM&(B@C_60_)-η^5^]_∞_ (TM = V, Cr) SMWs under external strains. For 1D [V&(B@C_60_)-η^6^]_∞_ SMW, it undergoes an AFM HM-AFM semiconductor (SC) transition under 5% and 10% compressive strain (see Figure 5a,b and Table 2). On the contrary, it is changed to a FM metal under 5% and 10% tensile strain (see Figure 5c,d and Table 2). In addition, 1D [Cr&(B@C_60_)-η^5^]_∞_ SMW transfers to both a FM metal and a ferrimagnetic (FIM) metal under 5% compressive stain and 5% (10%) tensile strain (see Figure 5f–h and Table 2), respectively, and changes to an AFM metal under 10% compressive strain (see Figure 5e and Table 2). 

## 4. Conclusions

Using first principles calculations, we systematically investigated the structure, electronic and magnetic properties of 3*d* transition metal atoms and B@C_60_ sandwich clusters, TM&(B@C_60_)_2_ (TM = V, Cr), and their 1D infinite SMWs, [TM&(B@C_60_)]_∞_. Our results showed that all the studied systems possess normal sandwich structures with extremely thermodynamic stabilities. It was found that respective η^6^- and η^5^-bonding configurations are confirmed for the systems with TM = V and Cr. One-dimensional [V&(B@C_60_)-η^6^]_∞_ and [Cr&(B@C_60_)-η^5^]_∞_ SMWs are an antiferromagnetic half-metal and a ferromagnetic metal. Furthermore, the magnetic properties can be modulated by exerting biaxial compressive and tensile strains. Finally, we should state that the diverse electronic and magnetic properties of the studied complexes may be highly sensitive to their surroundings [48,49]. Therefore, exploring their performance in a complicated environment, instead of non-free-standing states, is also of importance.

## Figures and Tables

**Table 1:** Optimized lattice constant (L, Å), local magnetic moment of TM atom and B atom (LMM, μ_B_), binding energy (E_b_, eV), the Bader charge (Δe, e) transferred from TM atom to B@C_60_ molecule, distance of TM atom to the mass center of faced C*_n_* rings (d_TM-C60_), distance of B atom to the mass center of the nearest η^5^- or η^6^-carbon ring in C_60_ (d_B-C60_, Å), deviations of the encapsulated B atom to the mass center of C_60_ molecule (Δ*h*, Å).

Sys	L(Å)	LMM(μ_B_)		E_b_(eV)	Δe(e)	*d*_TM-C60_(Å)	*d*_B-C60_(Å)	Δ*h*(Å)
		TM	B					
V&(B@C_60_)_2_-η^5^	——	3.00	0.41	−1.90	1.13	1.96	3.28–3.53	0.03
V&(B@C_60_)_2_-η^6^	——	1.00	0.41	−2.23	1.30	1.73	3.12–3.21	0.02
Cr&(B@C_60_)_2_-η^5^	——	6.00	0.42	−3.81	1.09	2.00	3.24	0.08
Cr&(B@C_60_)_2_-η^6^	——	2.00	0.41	−3.31	1.00	1.73	3.10–3.22	0.03
[V&(B@C_60_)-η^6^]_∞_	9.84	1.00	0.41	−5.24	0.32	1.69	3.46–3.40	0.32
[Cr&(B@C_60_)-η^5^]_∞_	10.64	4.00	0.41	−8.67	0.09	2.01	3.31–3.32	0.08

**Table 2:** Local magnetic moment of TM atom and B atom (LMM, μ_B_) under different strain.

Sys		LMM(μ_B_)			
		ε = −10%	ε = −5%	ε = +5%	ε = +10%
[V&(B@C_60_)-η^6^]_∞_	V	1.31/−1.33	1.00/−0.97	1.83/1.83	2.75/2.75
	B	0.36/−0.35	0.41/−0.42	0.42/0.42	0.42/0.42
[Cr&(B@C_60_)-η^5^]_∞_	Cr	2.85/−2.92	3.12/3.12	4.03/4.03	4.25/4.25
	B	0.38/0.38	0.37/0.37	−0.42/−0.42	−0.32/−0.32

## Supplementary Materials

The following supporting information can be downloaded at: https://www.mdpi.com/article/10.3390/nano12162770/s1, Table S1. Different spin states for each system. Table S2. The energy difference between FM and AFM states. Atomic coordination of V&(B@C60)_2_-η^5^. Atomic coordination of V&(B@C60)_2_-η^6^. Atomic coordination of Cr&(B@C60)_2_-η^5^. Atomic coordination of Cr&(B@C60)_2_-η^6^. Atomic coordination of [V&(B@C60)-η^6^]_∞_. Atomic coordination of [Cr&(B@C60)-η^5^]_∞_.
