# Supplementary material for: Ab Initio Study of Structural, Electronic and Magnetic Properties of TM&(B@C60) (TM = V, Cr) Sandwich Clusters and Infinite Molecular Wires
Source: Nanomaterials (Basel). 2022 Aug 12;12(16):2770. doi: 10.3390/nano12162770 (PMC9415518; doi:10.3390/nano12162770)
Supplement: Supplementary file 1 [file nanomaterials-12-02770-s001.zip › nanomaterials-1846840-supplementary.pdf]

nanomaterials

Supporting Information

Ab Initio Study of Structural, Electronic and Magnetic Properties of TM&(B@C_{60}) (TM = V, Cr) Sandwich Clusters and Infinite Molecular Wires

Jie Ji [1], Tianxia Guo [1], Liyan Qian [1], Xiaokang Xu [1], Huanning Yang [1], Yue Xie [1], Maoshuai He [2], Xiaojing Yao [3,*] Xiuyun Zhang [1,*] and Yongjun Liu [1,*]

1 College of Physics Science and Technology, Yangzhou University, Yangzhou 225002, China
2 College of Chemistry and Molecular Engineering, Qingdao University of Science and Technology, Qingdao 266042, China
3 College of Physics and Hebei Advanced Thin Film Laboratory, Hebei Normal University, Shijiazhuang 050024, China
* Correspondence: xjyao@hebtu.edu.cn (X.Y.); xyzhang@yzu.edu.cn (X.Z.); yjliu@yzu.edu.cn (Y.L.)

Table S1. Different spin states for each system, ΔE_1 is the energy difference between the different states and the magnetic ground states.

sys	MM(μ_B)	ΔE_1(eV)	sys	MM(μ_B)	ΔE_1(eV)
	1.00	0.94		4.00	0.07
V&(B@C_{60})$_2$_η^5	3.00	0.00	[V&(B@C_{60})$_2$_η^5]$_\infty$	6.00	0.00
	5.00	0.01		8.00	1.35
	1.00	0.00		2.00	0.00
V&(B@C_{60})$_2$_η^6	3.00	0.03	[V&(B@C_{60})$_2$_η^6]$_\infty$	4.00	0.07
	4.00	0.03		5.00	0.01
Cr&(B@C_{60})$_2$_η^5	6.00	0.00	[Cr&(B@C_{60})_η^5]$_\infty$	7.00	0.00
	8.00	0.41		9.00	1.38
	2.00	0.00		1.00	2.74
Cr&(B@C_{60})$_2$_η^6	4.00	0.17	[Cr&(B@C_{60})_η^6]$_\infty$	3.00	0.00
				5.00	1.01

Table S2. The energy difference between FM and AFM states (ΔE_2=E_{FM}-E_{AFM}, eV).

sys	coupling	ΔE_2
[V&(B@C_{60})$_2$_η^6]$_\infty$	AFM	0.35
[Cr&(B@C_{60})_η^5]$_\infty$	FM	-0.1

Atomic coordination of V&(B@C_{60})$_2$_η^5

V&(B@C_{60})$_2$_η^5

1.00000000000000

20.0000000000000000 0.0000000000000000 0.0000000000000000

0.0000000000000000 26.0000000000000000 0.0000000000000000

0.0000000000000000 0.0000000000000000 20.0000000000000000

B C V

2 120 1

Direct

0.4995368986640565 0.7110652753452844 0.4998032600672940

0.4997308534484114 0.2987454813609195 0.4914072190609476

0.6087588711790616	0.6324409376893598	0.3934222647985906
0.6522771405640693	0.6326726665433668	0.5025650509623536
0.6217642179741841	0.7256933535965313	0.3745402634588674
0.5492631928843460	0.6322573360299990	0.3556789687552079
0.5397356994579816	0.5753747794107804	0.5479015055919653
0.5784371784653027	0.6033459346351101	0.5944387830750055
0.6373875733462792	0.6802999541028311	0.6085924615610644
0.6567701343234386	0.7260297564527968	0.5773210249433467
0.4919713107889727	0.6030067520310273	0.3777540634388939
0.4321531289464632	0.6322376479163220	0.3634396565333256
0.4523621506254523	0.6797490339667781	0.3312567953638573
0.5448760971437393	0.6327131903251033	0.6457978905808972
0.5815735596876970	0.6803251324748191	0.6549808195006481
0.6214081453553172	0.7734160784554670	0.5913415861240586
0.3870869942548337	0.8022260431436786	0.5445242219656251
0.3544362459585322	0.7256925927001299	0.5969076190466347
0.3645837171530388	0.7729854808149742	0.4290179318914535
0.4423404499423976	0.8304649776276347	0.5224150565831714
0.4748050981866904	0.7260264550984211	0.6731916915425185
0.4392833108343175	0.6802055824543901	0.6641544705837933
0.3752574988644257	0.6324267803301180	0.5876594426549582
0.3490610950861650	0.6322790407387743	0.5222287143739150
0.5036763522765990	0.8305921688838729	0.5612385566845437
0.5594350502110330	0.8305860800432421	0.5148361746698535
0.5325032739808451	0.8304605537836225	0.4474235407425722
0.4746480349836526	0.6327130296888768	0.6502707470806530
0.4347091570957882	0.6033298260597669	0.6036324891117342
0.3812617897819299	0.6030574358935834	0.4699171797923712
0.3911741552983856	0.7731179235279274	0.6062801853447355
0.3476022058618741	0.7729154111237071	0.4970492394945414
0.3778640493518420	0.6800151753472221	0.6254009115164987
0.4503778271808086	0.7733643276322900	0.6439314822148330
0.4600990434702414	0.8304041600038199	0.4520602929114420

0.4219030388464905	0.8022864755977981	0.4061309708213294
0.3622370815728692	0.7255100051680150	0.3912217668287501
0.3427557733684284	0.6797660028306625	0.4224465981116332
0.5076614534039654	0.8026404205890323	0.6209333452814300
0.5675792608442000	0.7734622504166259	0.6362165002989895
0.5473389948311995	0.7260666049654882	0.6684169414980454
0.4548792396359329	0.7729637527223988	0.3539766255247277
0.4181410803796189	0.7254951160987009	0.3447638471092679
0.3781020825942434	0.6322663807179054	0.4084670679294890
0.6136581977835213	0.6033313919632458	0.4547907382842422
0.6454232910789544	0.6800106036801057	0.4028423256978153
0.6350948988204163	0.6326716282528148	0.5708390166570902
0.5576158555355198	0.5753383355148971	0.4772402277637293
0.5247959896800355	0.6797473876054596	0.3264939406176801
0.5602579718093870	0.7255392047372446	0.3357324574600187
0.6242948497464568	0.7731341022923298	0.4123365583591177
0.6504140367017797	0.7733145727179334	0.4774716878403291
0.4959801526229819	0.5752459156852497	0.4382647785114723
0.4399709674341615	0.5752587669615029	0.4848648878232633
0.4670104799684550	0.5753499789025291	0.5525914336889323
0.5248415609379452	0.7729086662227573	0.3496375607270665
0.5643228981479459	0.8022444105057518	0.3970866876043528
0.6173323937601495	0.8025769428740690	0.5296174303239464
0.6723517433432836	0.6801631904040724	0.4702990847370697
0.6747168010119330	0.7259857283741494	0.5068661538072758
0.3274695091223467	0.7255572866033307	0.5293584347216366
0.3248379666121058	0.6797614640617716	0.4928125689516070
0.6089240568530793	0.2265993281256986	0.3939189344360641
0.6524319955396222	0.2268038049990173	0.5029242758704910
0.6222134633256514	0.3198263944584279	0.3748107647076825
0.5497008999273465	0.2264537993066476	0.3564808880979255
0.5398300197798891	0.1691374462339396	0.5479681160840545
0.5778021446045367	0.1975891175667442	0.5936500766804504

0.6374990311850017	0.2743979390579449	0.6086045203626494
0.6571963919265250	0.3201686317472037	0.5775688674598640
0.4924255416846455	0.1971211841491988	0.3794733897700725
0.4325620718776853	0.2263400479771373	0.3641244835595989
0.4527878148004653	0.2737591713723266	0.3319658729172167
0.5448896529647285	0.2269111292184281	0.6458557240588491
0.5816097742087130	0.2743907873490178	0.6550770077395435
0.6218931435711827	0.3677052751151788	0.5915971553623121
0.3863454606371977	0.3966400554995649	0.5453624757984881
0.3544957722916169	0.3198278067358974	0.5973462763819812
0.3650679939773649	0.3671817376987128	0.4295189351874060
0.4423684998634796	0.4247696557199332	0.5229374686006659
0.4750808426361201	0.3200762892347283	0.6735211325543581
0.4396180991524612	0.2742275437598827	0.6643974641345284
0.3757341486580129	0.2266180275858010	0.5877865970394088
0.3497560676353508	0.2264837985275415	0.5227220945652666
0.5039885579470135	0.4248240412448272	0.5618270416718666
0.5600449206701971	0.4248619916447239	0.5152332214004898
0.5330685840933337	0.4248372469346512	0.4475452028637883
0.4749832396955271	0.2268271646666609	0.6504459821138635
0.4356465385848167	0.1974228952690515	0.6029697396030749
0.3828707931555155	0.1971727871732966	0.4706248651190518
0.3912081021830158	0.3674255185422811	0.6066638098668353
0.3477091249333875	0.3672544798856263	0.4977064786825158
0.3781745282629437	0.2741067669938251	0.6255744585457603
0.4506720662131479	0.3675434258369973	0.6442538907974090
0.4603429024480896	0.4247665320899690	0.4522983742492289
0.4218224526265965	0.3964990144630818	0.4060025845810825
0.3627692924446916	0.3195718659913765	0.3917533353498355
0.3433724886963042	0.2737771532716081	0.4229627234006519
0.5080008900564572	0.3968444475732166	0.6221203660187037
0.5677790931447098	0.3676981767626712	0.6366151472989486
0.5475178641291942	0.3201653101649581	0.6687691428941135

0.4552880763579544	0.3671894787708915	0.3545027961238604
0.4185938058549783	0.3195678342876582	0.3453633431046327
0.3787176933242196	0.2263702741445585	0.4089725908952250
0.6129415180250611	0.1973919688179910	0.4555797237719449
0.6456447725960258	0.2740869115929155	0.4032281558479054
0.6351312926238519	0.2269119321343429	0.5708084878501745
0.5577129484101772	0.1690417339306559	0.4776676207410086
0.5253196178974487	0.2738338606674159	0.3272222229694324
0.5608479971879290	0.3197013805998090	0.3360672343629925
0.6247095545428303	0.3674474984577801	0.4125523455604155
0.6508342143096192	0.3675618736317785	0.4778801394507252
0.4963684269896700	0.1689198543263255	0.4389375047901096
0.4405846241646731	0.1689373588802221	0.4853244719036076
0.4674389869409979	0.1690618246568829	0.5527025101823712
0.5255140448368983	0.3672694555831167	0.3499085228807791
0.5653090825623228	0.3966563781104888	0.3965798962744333
0.6185929068421843	0.3968673166918899	0.5301944797872408
0.6726101230722207	0.2742072326451073	0.4707084555165688
0.6751077981760900	0.3200756593294278	0.5072232510542509
0.3275998041371989	0.3197104546708583	0.5299370433112719
0.3254248721557390	0.2738547665134217	0.4933949308113167
0.5000065114340706	0.5000711534071192	0.5000821828654587

Atomic coordination of V&(B@C_{60})$_2$_η^6

V&(B@C_{60})$_2$_η^6

1.00000000000000

20.0000000000000000	0.0000000000000000	0.0000000000000000
0.0000000000000000	20.0000000000000000	0.0000000000000000
0.0000000000000000	0.0000000000000000	26.0000000000000000

C	B	V
120	2	1

Selective dynamics

Direct

0.3454967786866974	0.4635154847639805	0.6293515993069613
0.3330825474416051	0.5589689410896059	0.6814933529926724
0.3653443783559263	0.3844428939752933	0.7009288567170793
0.3901931877919189	0.4278573494036297	0.5983854515345729
0.4247234442303947	0.6307595004166585	0.6183910576353113
0.4123296267335322	0.6522678853133559	0.6688216932345004
0.3915657554037509	0.6154892606717878	0.7530089257624766
0.3839554678138447	0.5588512852499228	0.7840873387703187
0.4376015016967082	0.4644630233144322	0.5667085700120522
0.5003709417001936	0.4281672798058495	0.5667102723458920
0.4922493536546545	0.3688222125323691	0.5983769782333044
0.4673581508071205	0.6744092366895336	0.7009274023028731
0.4545743291845616	0.6517746734712907	0.7529957196260317
0.4387017142850040	0.5363229288691124	0.8163726201548686
0.6542900245050400	0.4634883541541127	0.7529643178583727
0.6675959626894640	0.5585675105781902	0.7008891049411683
0.6344040999103049	0.3846358358391835	0.6814342012357447
0.6089821162953837	0.4286588265997482	0.7840559921461699
0.5751616485570681	0.6297762186893343	0.7640009194180385
0.5876162679032885	0.6512952797096615	0.7136910562164686
0.6091668271052637	0.6154214799942407	0.6293085578051194
0.6175938624699063	0.5588566017282265	0.5983378203526944
0.5621628501017444	0.4648750980720059	0.8163593489551142
0.4993032348359814	0.4286591900765264	0.8163793751063515
0.5071419920292787	0.3699795818614262	0.7840753974116457
0.5328064014781602	0.6740253426191199	0.6814706954195739
0.5457623877035210	0.6520999631690485	0.6293287456055181
0.5621233462099049	0.5361956615949497	0.5666725233804876
0.6543479829379735	0.5362133548309896	0.7529656802377166
0.6674708019576632	0.4411099262629899	0.7008814756630593
0.6346306427668678	0.6151248341919731	0.6814457570126150
0.6091092048078496	0.5711021655023953	0.7840417430982793
0.5749291322120369	0.3700496337567708	0.7640073214515410

0.5873285846761760	0.3485264644646301	0.7136750931806141
0.6089179055697070	0.3843865081546891	0.6293108879715530
0.6174645285576293	0.4409392882207607	0.5983415801756807
0.5622234262643003	0.5349775643444843	0.8163456966926061
0.4994268665102037	0.5712938806358904	0.8163514066116579
0.5073924498046899	0.6299493121260580	0.7840581118576780
0.5324738594999675	0.3259168917924947	0.6814503575722061
0.5454642351824361	0.3478470548007352	0.6293250739483545
0.5620366899737191	0.4636845285284575	0.5666934305008295
0.3455587492523721	0.5367602127450608	0.6293573561309083
0.3329625848316665	0.4413374080086989	0.6814841156516592
0.3655736782019484	0.6157763691015355	0.7009394475478458
0.3903226091441400	0.5723576263083638	0.5983804553910953
0.4244857685657346	0.3693894010457723	0.6183898322972371
0.4120432455860680	0.3478978787638022	0.6688016639032645
0.3913160235770318	0.3846788159163487	0.7530149541874219
0.3838299880496602	0.4413212423055268	0.7840916607927927
0.4376663704078392	0.5356529395896599	0.5667108436865003
0.5004978739530922	0.5718483276740640	0.5666922803788758
0.4924973543190062	0.6312239286670088	0.5983684540234786
0.4670222233348925	0.3256344289527691	0.7009088617777950
0.4542762880701434	0.3482540527286155	0.7530001769560420
0.4386150956830374	0.4637678722947406	0.8163873067674678
0.3252486885786676	0.5001532050846776	0.7137267464507051
0.3501010859431722	0.5001334646455788	0.7640392286898294
0.6757992999648278	0.4998363308475894	0.6687604544715661
0.6509741379641946	0.4998551255429914	0.6183485321359933
0.3455647134949297	0.4637262718072243	0.2470075350305917
0.3324568434131677	0.5589215329788532	0.2990718397657369
0.3652367559266965	0.3846617042504543	0.3184793282955516
0.3908469295684808	0.4287956352444495	0.2159787471524534
0.4251396694413878	0.6299359696481299	0.2360589011432277
0.4126830954695678	0.6515803388905406	0.2863076443757763

0.3910801467498799	0.6157413912723251	0.3705783635870433
0.3825470513234233	0.5590951274707268	0.4014532743708776
0.4377541715778793	0.4649726575561174	0.1837067896917966
0.5005575364850001	0.4286316196310024	0.1837093672125622
0.4925922459126776	0.3699487615611421	0.2159925554841895
0.4675945933414107	0.6741853165893384	0.3184662963515705
0.4545600283689248	0.6523149496026414	0.3705786747232198
0.4380311283064742	0.5363217741622158	0.4330656062324715
0.6545441587755932	0.4632504350103983	0.3705462699675297
0.6671462260388964	0.5587664585355567	0.3184338263670319
0.6345089485147299	0.3841138978893442	0.2990258141636203
0.6097162242332338	0.4276125389564622	0.4014416856439471
0.5755961687260746	0.6307298472399158	0.3814879485551304
0.5880639936012746	0.6522723035154568	0.3311183935484893
0.6087733773556576	0.6154938144024574	0.2469878149778671
0.6161799057976576	0.5587183768449906	0.2160101053127028
0.5623294029425934	0.4643773052995109	0.4330526147996200
0.4994969112768238	0.4281522381418758	0.4330236066866634
0.5074522687675606	0.3687318613519901	0.4013946513038202
0.5330418079791276	0.6744753225867031	0.2990606866179854
0.5458266157026006	0.6518797771400749	0.2470083423258531
0.5613748551067493	0.5362339116571514	0.1837453695314336
0.6546321838575923	0.5365421811957971	0.3705572964298622
0.6670510126145102	0.4409915986283264	0.3184276524511926
0.6347776750376309	0.6156968913159288	0.2990382244048531
0.6099272714665441	0.5722748001917872	0.4014931794861260
0.5752377586561784	0.3692337822180396	0.3814356350403135
0.5876923468788599	0.3476307926802801	0.3310836517429964
0.6084763658796952	0.3843899502526261	0.2469782001925843
0.6160552367377260	0.4411070161805176	0.2159921755436696
0.5624456783629788	0.5355716989109052	0.4330932147794306
0.4996736735512414	0.5718975702669482	0.4331007736199913
0.5078078685702080	0.6313322433125220	0.4014766533038033

0.5325868128476053	0.3255325367078410	0.2990523508404039
0.5454291463724935	0.3481159523846501	0.2470012684491759
0.5612851618126523	0.4636581321472718	0.1837294390701509
0.3456359427678171	0.5364879621520502	0.2470070940267812
0.3323624580282049	0.4413081224037868	0.2990743290404393
0.3655291102011842	0.6155032080108073	0.3184746699166596
0.3910271254877774	0.5713381040161072	0.2160124010222738
0.4248105563395039	0.3701311411582143	0.2360361398394292
0.4123131310157003	0.3485282155659183	0.2862833888734279
0.3907372436960646	0.3843208046083009	0.3705894526210257
0.3824172499488319	0.4410483479482449	0.4014379082066910
0.4378547694909665	0.5350914031487924	0.1837331177459660
0.5007263146534037	0.5713331586548235	0.1837543473612299
0.4929196841109314	0.6300246776183251	0.2160461659860586
0.4671383848989112	0.3258070430484483	0.3184641943122766
0.4541274824150119	0.3476721107426350	0.3705727026276050
0.4379326035143261	0.4637748413116592	0.4330330210026723
0.3240942713675395	0.5001269583928317	0.3311217396673029
0.3490451782846248	0.5000996473177809	0.3814982754150163
0.6749030088178706	0.4998677333105197	0.2862557114315408
0.6499214371984368	0.4998860344701603	0.2359958294812865
0.5003095099518479	0.4995973729106169	0.6900297705156480
0.4987114955393736	0.5006037524414246	0.3132719896203951
0.5000335717837068	0.5000275505827960	0.5000382132814487

Atomic coordination of Cr&$(B@C_{60})_2$_η^5

Cr&$(B@C_{60})_2$_η^5

1.00000000000000

20.0000000000000000	0.0000000000000000	0.0000000000000000
0.0000000000000000	26.0000000000000000	0.0000000000000000

0.0000000000000000 0.0000000000000000 20.0000000000000000

B C Cr

2 120 1

Selective dynamics

Direct

0.4973879419741890 0.7016985931967843 0.5010642324618884

0.4985961067382378 0.2983190239647975 0.4988514257790194

0.6089623443800941 0.6338661154563089 0.3932452019337418

0.6525941212566173 0.6341711169639414 0.5023585756905536

0.6217720497695373 0.7271882229180525 0.3743025366069700

0.5494221549397684 0.6336922053858746 0.3557364434900900

0.5398585056480505 0.5771282968343768 0.5477976241945604

0.5785311521199381 0.6049760291869408 0.5942137143306824

0.6375080906945451 0.6818904239957967 0.6082397250222589

0.6567830879283921 0.7276906213630195 0.5769006245067703

0.4920844640668064 0.6043966307010414 0.3779922350968041

0.4321571141390678 0.6335232328256072 0.3635000043990637

0.4524003239882218 0.6809989089356274 0.3313409541867339

0.5450186326577122 0.6343064153484020 0.6457623580183521

0.5816941649557360 0.6819044518433655 0.6547128557741063

0.6213798787787939 0.7750774289184017 0.5908491954163106

0.3870804502084780 0.8038869986616540 0.5443940634463179

0.3544552099112428 0.7272633069887896 0.5969279180415787

0.3647661368470125 0.7743212792775586 0.4292100101379077

0.4422407873005990 0.8322551541885426 0.5222051230298319

0.4749078325129453 0.7276167431586312 0.6727946977749776

0.4394015981042860 0.6817020504231976 0.6641292519895030

0.3753692227685814 0.6339107169411748 0.5877964353788060

0.3492446199074967 0.6337292678967948 0.5224546420851158

0.5036193734036598 0.8324277256421397 0.5608623920500294

0.5593568482025348 0.8324123668624690 0.5144420804607142

0.5324332871391970 0.8322244654167705 0.4470939125996800

0.4748059985539554 0.6342045242268627 0.6504169711325750

0.4349245039174481	0.6047695590844981	0.6037346589877842
0.3814803605708814	0.6044016154170549	0.4700965338879395
0.3911448402278193	0.7747523070487908	0.6061093304208689
0.3475611788634864	0.7744471402255880	0.4971285364868611
0.3779485926430357	0.6815223365284877	0.6254229894185784
0.4504349007769761	0.7749247463222062	0.6434531818538768
0.4600529333981974	0.8321237471965676	0.4518919661745066
0.4220567226480485	0.8036368644320667	0.4062802872337513
0.3624174274809743	0.7268218999508697	0.3914698726691601
0.3428242569171778	0.6810250280145540	0.4226149855218277
0.5076400186505965	0.8042377465716580	0.6203265988285374
0.5675532042233694	0.7750965337603810	0.6357015575030139
0.5474106190587457	0.7277200601980685	0.6679966098062826
0.4549567392946358	0.7742974222997285	0.3540971416000603
0.4182507125596509	0.7268101085996047	0.3449667326334386
0.3780989745557218	0.6335317111607706	0.4085464190620658
0.6138673524066842	0.6047411724812722	0.4546950792983710
0.6455542448362305	0.6814586935418386	0.4025630057863407
0.6353155098789808	0.6342877382720820	0.5705593009683622
0.5577301030074910	0.5770120355175865	0.4772785928149110
0.5248718838047604	0.6811012852655647	0.3265373261904849
0.5602889515731909	0.7270065594189827	0.3355739599838172
0.6241911343473349	0.7746911219339788	0.4120164181983759
0.6502042624007546	0.7748756187182608	0.4770774981584069
0.4961625855119701	0.5768218641411857	0.4384863155152490
0.4402343523635159	0.5768033702492018	0.4850584491724668
0.4672598235738995	0.5770028345128793	0.5526199002756141
0.5248718331417599	0.7743927378552826	0.3494666646366682
0.5642221909673580	0.8038331488190794	0.3968645036124193
0.6171094275085092	0.8042076161815150	0.5291447474539888
0.6725259539713330	0.6816487035939321	0.4699938700990813
0.6746333660853524	0.7275670652729784	0.5064704416454349
0.3274722603532881	0.7270724725130930	0.5294667853202589

0.3249898792634200	0.6811500161577071	0.4930157564193615
0.6089141662922298	0.2252594964843917	0.3938992382464270
0.6524989120227414	0.2255731238790429	0.5028780233849747
0.6221262638854805	0.3184845979968423	0.3745693078753750
0.5496280986254998	0.2250736697150301	0.3565521789671897
0.5400001717342457	0.1678912152240288	0.5481135534302887
0.5780024051544111	0.1963812117986433	0.5937236535671607
0.6376522849733937	0.2732009001131031	0.6085330012828496
0.6572563563494935	0.3189937112763287	0.5773900604490396
0.4924222909335408	0.1957581400855077	0.3796809864089808
0.4325110523173685	0.2248932344424076	0.3643092522024224
0.4526594453965009	0.2722698886674614	0.3320145380900160
0.5451004711468463	0.2257208534314757	0.6459021188958273
0.5818101850795719	0.2732116049006568	0.6550287944246552
0.6219672115451247	0.3664795355815129	0.5914519179737412
0.3861886065870229	0.3952696689718163	0.5453008649469642
0.3545233279148223	0.3185415157200244	0.5974279739210104
0.3647478579774618	0.3657075574251289	0.4294345641309121
0.4423235164934323	0.4230083112516559	0.5227160354144008
0.4751898769475669	0.3189073619173659	0.6734560092640396
0.4397692180149080	0.2729969070883480	0.6644331073493828
0.3758682027654269	0.2253037593885559	0.5879855111969644
0.3498546504917711	0.2251105586649479	0.5229242015599197
0.5038932052930839	0.4231821613145214	0.5615050846803363
0.5598163957886869	0.4231770945565652	0.5149346498785030
0.5327959299843880	0.4229812870987471	0.4473786640386958
0.4751857840099296	0.2256128415417538	0.6505399454692974
0.4358359724483949	0.1961667203823988	0.6031422747359816
0.3829460610707847	0.1957771076838551	0.4708587610687798
0.3911010810951885	0.3661435220016530	0.6067481974848732
0.3474699935147978	0.3658291389747415	0.4976380618642036
0.3782915615446415	0.2728100336148925	0.6256974963184202
0.4506413101195041	0.3663153451854121	0.6442573125834421

0.4601972438113772	0.4228839771675020	0.4521941323681060
0.4215301530053018	0.3950259971207089	0.4057760717283871
0.3625629511150208	0.3180956571363953	0.3917673870555526
0.3432750972424413	0.2722888135684937	0.4230981392996513
0.5079777601690499	0.3956107165613447	0.6219971502188623
0.5679053846940394	0.3664906593918920	0.6364890141741443
0.5476613382643558	0.3190178878085655	0.6686552021817369
0.4550501653971469	0.3656894838206581	0.3542370925657240
0.4183779213486500	0.3180853550957631	0.3452952778040286
0.3786778223237265	0.2249040928580132	0.4091535262193546
0.6129758491536521	0.1961305591691127	0.4556159601973448
0.6456058530990902	0.2727515837006378	0.4030774305384916
0.6352980184526280	0.2256989889875705	0.5707958943107041
0.5578113837838210	0.1677593296808243	0.4778023158726971
0.5251612746354787	0.2723783900736593	0.3272064750837559
0.5606712595498448	0.3182949627917664	0.3358650454985992
0.6247001031665008	0.3660936508199004	0.4122030468100839
0.6508214396739423	0.3662769275499979	0.4775419232664688
0.4964350274231115	0.1675700551954525	0.4391449022255360
0.4406962768009152	0.1675773872565264	0.4855630374760009
0.4676200568702825	0.1677763966742746	0.5529113036718440
0.5252631608842813	0.3657897534194718	0.3495764339408178
0.5651386828712018	0.3952245707175366	0.3962648861585486
0.6185748701402877	0.3955970516066300	0.5298972828665623
0.6725924936711385	0.2729476408978757	0.4705382393282607
0.6750906261920947	0.3188658450635824	0.5069883739724669
0.3275550669627587	0.3183432947487582	0.5299985570906858
0.3254280396267871	0.2724165073660398	0.4935269297804717
0.4999622835247982	0.5000041220888310	0.4999903994012323

Atomic coordination of Cr&(B@C_{60})$_2$_η^6

Cr&(B@C_{60})$_2$_η^6

1.00000000000000

20.0000000000000000	0.0000000000000000	0.0000000000000000
0.0000000000000000	20.0000000000000000	0.0000000000000000
0.0000000000000000	0.0000000000000000	26.0000000000000000

C B Cr

120 2 1

Selective dynamics

Direct

0.3456411772130888	0.4635801439536998	0.6292303851965397
0.3332117448358811	0.5589030162633599	0.6814481439979777
0.3654456308995219	0.3845256588232306	0.7008390659438077
0.3901675651042825	0.4278627057929653	0.5980974106694646
0.4247878108871666	0.6306807519484438	0.6182164439215908
0.4124116169595097	0.6521533335748573	0.6686866993821322
0.3916086879146579	0.6154472932765996	0.7529889454776476
0.3838656606743138	0.5588169818058167	0.7841288070996856
0.4378192693803845	0.4646308898308580	0.5663906647968946
0.5003663182877710	0.4284345576508368	0.5663748900105692
0.4922449919055068	0.3688149837603876	0.5980918616221143
0.4673799197654924	0.6742775514404176	0.7008091943347752
0.4545661787776771	0.6517309707453629	0.7529699131320799
0.4387020339433535	0.5362863842118394	0.8163251197197897
0.6542207306261969	0.4635028796702794	0.7529589442342872
0.6674391392868543	0.5585386473520492	0.7007827003359809
0.6342548632777802	0.3847243505524804	0.6814127883492075
0.6089841494604712	0.4285576144153919	0.7840995311342915
0.5751963535174041	0.6298701929228621	0.7640439248738073
0.5875949870722996	0.6513118990266460	0.7136266966879357
0.6089730145476210	0.6152564852813289	0.6291974216779509
0.6175854648625954	0.5588782084799869	0.5980379485846813
0.5621093088799152	0.4648722625381689	0.8163039353909826
0.4992738864188689	0.4286802938817774	0.8163086566737382
0.5071986148638310	0.3699274384957093	0.7841079210126224
0.5327989829241454	0.6738972352665621	0.6814168721134055

0.5456681531175921	0.6518936675238974	0.6292051857155436
0.5618868100535475	0.5361061738829819	0.5663686087290430
0.6542998848976339	0.5361791502698650	0.7529487624538723
0.6673370138879380	0.4411606718342815	0.7007895402668890
0.6344933355180677	0.6150673591874223	0.6814026925967450
0.6090986121973181	0.5711741741376873	0.7840600198139058
0.5749587291114843	0.3699284868043024	0.7641019886986000
0.5872616526622298	0.3485876814956322	0.7136556401488606
0.6087750631692860	0.3845503602103202	0.6291896259013476
0.6174840894004477	0.4409428044687576	0.5980251504203425
0.5621666974900972	0.5349305237760514	0.8162838335472622
0.4994077294726695	0.5712463782706243	0.8162958807175249
0.5074336370156329	0.6299959439581142	0.7840705768165481
0.5324389358789994	0.3260883979095575	0.6814350179729803
0.5453973624965907	0.3480207702393472	0.6292076923305565
0.5618317901052690	0.4638424199127391	0.5663512994640559
0.3457174104563203	0.5367451767945494	0.6292204399782814
0.3331102951207568	0.4414044903383056	0.6814555144320690
0.3656719008015714	0.6157014878511406	0.7008282552843710
0.3902866836711675	0.5724072852960784	0.5980595354561737
0.4245438403712245	0.3695114377707217	0.6182622902240072
0.4120752439056257	0.3479516460839617	0.6687096521537926
0.3914089254270339	0.3847431595688212	0.7529869423445124
0.3837583262003754	0.4413615875840360	0.7841275814874743
0.4378840984929092	0.5355599023851348	0.5663863978817166
0.5005011608312450	0.5716241501234891	0.5663873130311986
0.4924861264330582	0.6312575604069443	0.5980667584808077
0.4670217847921254	0.3257644061020211	0.7008262091809589
0.4542909066439131	0.3483598602724714	0.7529786204949475
0.4386432456676608	0.4637600093057138	0.8163184364202134
0.3253062211835036	0.5001545100799210	0.7136955138025426
0.3499593207210342	0.5001166790881677	0.7641335506057908
0.6756452955572269	0.4998490777083755	0.6686414935463439

0.6508542404969818	0.4998836090906822	0.6181735082822885
0.3455570692428269	0.4637882838619202	0.2469956782736234
0.3324882952176783	0.5588482086498161	0.2991406817746290
0.3654001729509193	0.3849579855514776	0.3184873150731523
0.3908361736543794	0.4288375136562849	0.2159659526476680
0.4250346284613714	0.6299960148094864	0.2359545589447240
0.4126536119659500	0.6514780272973494	0.2862997665229466
0.3911758279268730	0.6154491450025746	0.3706991560777205
0.3824090240217267	0.5590870332268220	0.4017967830282123
0.4377806605977583	0.4650460265738678	0.1837358920426560
0.5005763979621464	0.4287273307262440	0.1837063752064959
0.4925414487032190	0.3699697198001004	0.2159094495377848
0.4675620362614052	0.6740071431625664	0.3184911399840257
0.4546101022175735	0.6520070862901375	0.3706997854562153
0.4381683634846955	0.5362022207277954	0.4334407632574597
0.6545042996920613	0.4632976184876363	0.3707002309353447
0.6670842876591945	0.5586456151495851	0.3184758038123152
0.6343947785442702	0.3843568970263677	0.2991180446256263
0.6097529777397863	0.4276930538485812	0.4016730446374316
0.5755714125371977	0.6305830196686629	0.3816517913753031
0.5879918746652680	0.6520684812077284	0.3312458148088629
0.6086923563212698	0.6153062737490151	0.2469813484488852
0.6162562198998871	0.5586526935209181	0.2159240936675738
0.5621257345927431	0.4645434333990981	0.4333290877986724
0.4994553129003576	0.4284440646949222	0.4333587667267554
0.5074703045492274	0.3687352173989126	0.4017302373142365
0.5330077433310253	0.6743326344378752	0.2991187520739850
0.5457934324360378	0.6517377354422977	0.2469707707883612
0.5613606950117231	0.5362168914733931	0.1837017970696871
0.6545681949307570	0.5364800957786517	0.3707055350731031
0.6669952679322250	0.4410914224777929	0.3184727373376717
0.6346454857190734	0.6154436340014272	0.2991298001792146
0.6099400978465410	0.5721711732000262	0.4017181666575048

0.5752483642576453	0.3693362867472548	0.3816146651816744
0.5876598104574527	0.3477883136761904	0.3312232506551480
0.6084132895024230	0.384564253009118 7	0.2469780066937257
0.6161368954875296	0.4411682353037279	0.2159101045848628
0.5622231490289539	0.5354029953908579	0.4333629543438419
0.4996294790406581	0.5715890202006289	0.4334059941995267
0.5077979881962313	0.6313104622694629	0.4017960119322149
0.5325910001216100	0.3256584487837880	0.2991112183860308
0.5454186762353186	0.3482517230437909	0.2469723029178621
0.5612720664710378	0.4636888964854646	0.1836944080435536
0.3456144264066688	0.5364631043519076	0.2469904598648281
0.3324000417600275	0.4414298294457528	0.2991447322886316
0.3656542201676117	0.6152501599627871	0.3184904417655757
0.3910022897066935	0.5713303181517475	0.2159949907831486
0.4247313799489449	0.3701163519382175	0.2359342264659092
0.4123240680448197	0.3486770703342942	0.2862875338450417
0.3908705755457814	0.3846598560267059	0.3707114956061430
0.3822979472792272	0.4410961332934277	0.4017760412128793
0.4378685893903287	0.5350398253780728	0.1837616462566339
0.5007429353732019	0.5712642546740313	0.1837397488711119
0.4928450587723484	0.6300240519915854	0.2159530552450469
0.4671437056247698	0.3260074351906914	0.3184850385243455
0.4542079444557162	0.3479876714136172	0.3707076409853190
0.4380730488545996	0.4639089702710305	0.4334275201208795
0.3241401993713222	0.5001450891757631	0.3312664549814211
0.3490272882004870	0.5001133291831540	0.3816583519190761
0.6748742369136927	0.4998614245389447	0.2862760890561213
0.6500412519182398	0.4998882003325772	0.2359362888284316
0.5003183055690533	0.4996420532165760	0.6903465665171603
0.5001199430653041	0.4999241133734671	0.3141915662224467
0.4999449824053680	0.5000181113854479	0.4997837697527755

Atomic coordination of [V&(B@C_{60})$_2$_η^6]$_\infty$

[V&(B@C_{60})$_2$_η^6]$_\infty$

1.00000000000000

20.0000000000000000	0.0000000000000000	0.0000000000000000
0.0000000000000000	20.0000000000000000	0.0000000000000000
0.0000000000000000	0.0000000000000000	19.6799999999999997

B	C	V
2	120	2

Selective dynamics

Direct

0.5078638436231975	0.5159960000000012	0.2081218770612458
0.4843780122772500	0.5159960000000012	0.7083201700012230
0.3416115181408728	0.4793919438026148	0.1151902058391625
0.3287527214092310	0.5748018264118647	0.1838814817938724
0.3615094871620311	0.4003847740564716	0.2096577950268973
0.3865518063428768	0.4438104307833743	0.0745459849919703
0.4208883334534383	0.6465235217063703	0.1009681331865746
0.4083791623095142	0.6680869714255796	0.1674408212430690
0.3871027300375943	0.6315711888426577	0.2783623019588286
0.3786738604300849	0.5749212814156791	0.3190750350094702
0.4339636157394066	0.4804214548363000	0.0330028249325598
0.4967382697530071	0.4442618807862571	0.0331482056762329
0.4886261260022254	0.3849172684782213	0.0746250981044893
0.4633925966642561	0.6904071027987645	0.2096594214481531
0.4505006852452400	0.6681569748108163	0.2783335568581008
0.4340278411742222	0.5522380764319816	0.3606779843051923
0.6507203490278738	0.4793927578853954	0.2783152885084415
0.6636778962583525	0.5748141347909312	0.2096376476251482
0.6309166725008216	0.4003441399571023	0.1838924668478382
0.6057595535243873	0.4438292540808015	0.3190262180383921
0.5714576367319735	0.6465312889524157	0.2926153388052789
0.5840056212818285	0.6680849287288391	0.2261071490140249

0.6052930023253725	0.6315790771249005	0.1152063115565417
0.6137780434457294	0.5749308653618497	0.0745123725809383
0.5584689298539302	0.4803724402616809	0.3607702980212071
0.4956718030234117	0.4441857565702668	0.3606175597294072
0.5037522418287436	0.3849033060106427	0.3190151213366661
0.5290179651367067	0.6904494220262530	0.1838995238366224
0.5419009919045664	0.6681773391610883	0.1152342767878174
0.5583712414102443	0.5522360859750036	0.0329895589845932
0.6507203490278738	0.5525992421146070	0.2783152885084415
0.6636778962583525	0.4571778652090724	0.2096376476251482
0.6309166725008216	0.6316478600428936	0.1838924668478382
0.6057595535243873	0.5881627459191936	0.3190262180383921
0.5714576367319735	0.3854607110475871	0.2926153388052789
0.5840056212818285	0.3639070712711643	0.2261071490140249
0.6052930023253725	0.4004129228751017	0.1152063115565417
0.6137780434457294	0.4570611346381462	0.0745123725809383
0.5584689298539302	0.5516195597383213	0.3607702980212071
0.4956718030234117	0.5878062434297349	0.3606175597294072
0.5037522418287436	0.6470886939893598	0.3190151213366661
0.5290179651367067	0.3415425779737493	0.1838995238366224
0.5419009919045664	0.3638146608389073	0.1152342767878174
0.5583712414102443	0.4797559140249991	0.0329895589845932
0.3416115181408728	0.5526000561973814	0.1151902058391625
0.3287527214092310	0.4571901735881377	0.1838814817938724
0.3615094871620311	0.6316072259435239	0.2096577950268973
0.3865518063428768	0.5881815692166275	0.0745459849919703
0.4208883334534383	0.3854684782936325	0.1009681331865746
0.4083791623095142	0.3639040285744182	0.1674408212430690
0.3871027300375943	0.4004208111573455	0.2783623019588286
0.3786738604300849	0.4570707185843235	0.3190750350094702
0.4339636157394066	0.5515705451637097	0.0330028249325598
0.4967382697530071	0.5877301192137454	0.0331482056762329
0.4886261260022254	0.6470747315217807	0.0746250981044893

0.4633925966642561	0.3415848972012389	0.2096594214481531
0.4505006852452400	0.3638350251891866	0.2783335568581008
0.4340278411742222	0.4797539235680208	0.3606779843051923
0.3206283499940339	0.5159960000000012	0.2261211202618808
0.3453161418468369	0.5159960000000012	0.2926894632686055
0.6718786747147822	0.5159960000000012	0.1674077575478010
0.6471359520275219	0.5159960000000012	0.1008741273085947
0.3414429065753167	0.4793985389643406	0.6152243249838906
0.3286114085047863	0.5748246784083531	0.6839102166704973
0.3616422150473305	0.4004570728244411	0.7096706192627098
0.3864032298262273	0.4438182344806817	0.5746286018332992
0.4208075272175347	0.6465291648263563	0.6009545619533314
0.4084726780226147	0.6680442919265084	0.6674603891080574
0.3874551656845996	0.6315350252907116	0.7783193837297541
0.3791449858495778	0.5748690338531801	0.8190125233493765
0.4337696310264427	0.4804568054174221	0.5331393864235144
0.4965011257510886	0.4441737197201396	0.5329448322408887
0.4884974297995682	0.3848616409849808	0.5745216364682613
0.4635224879043444	0.6904009832334217	0.7096651951582725
0.4508292675733870	0.6681809183127684	0.7783682307636759
0.4344781995626666	0.5522420137091995	0.8607343844911249
0.6510195426490966	0.4793979436675131	0.7782986873417044
0.6638164438949585	0.5748078748716358	0.7096035688502227
0.6309532565788812	0.4004093598492821	0.6838540197593093
0.6061671302417962	0.4437939563465715	0.8189918774987265
0.5718167255007307	0.6465779942736908	0.7926634496407818
0.5841157188853353	0.6680254377501806	0.7260997795947285
0.6052274920590397	0.6315849048212324	0.6151842643110069
0.6135652165905662	0.5749084082421958	0.5745453509612848
0.5588651674256787	0.4804239367968420	0.8605739913904834
0.4961436767232618	0.4441118461864820	0.8607632573021644
0.5041324132301830	0.3848438658399567	0.8191043345295611
0.5290952034349389	0.6903929941837166	0.6838818936517124

0.5418221062373739	0.6681690242405625	0.6151872611991400
0.5581074405437736	0.5522143383276853	0.5330545883089580
0.6510195426490966	0.5525940563324894	0.7782986873417044
0.6638164438949585	0.4571841251283665	0.7096035688502227
0.6309532565788812	0.6315826401507136	0.6838540197593093
0.6061671302417962	0.5881980436534242	0.8189918774987265
0.5718167255007307	0.3854140057263038	0.7926634496407818
0.5841157188853353	0.3639665622498224	0.7260997795947285
0.6052274920590397	0.4004070951787698	0.6151842643110069
0.6135652165905662	0.4570835917577997	0.5745453509612848
0.5588651674256787	0.5515680632031613	0.8605739913904834
0.4961436767232618	0.5878801538135198	0.8607632573021644
0.5041324132301830	0.6471481341600458	0.8191043345295611
0.5290952034349389	0.3415990058162858	0.6838818936517124
0.5418221062373739	0.3638229757594326	0.6151872611991400
0.5581074405437736	0.4797776616723172	0.5330545883089580
0.3414429065753167	0.5525934610356551	0.6152243249838906
0.3286114085047863	0.4571673215916487	0.6839102166704973
0.3616422150473305	0.6315349271755548	0.7096706192627098
0.3864032298262273	0.5881737655193204	0.5746286018332992
0.4208075272175347	0.3854628351736470	0.6009545619533314
0.4084726780226147	0.3639467080734891	0.6674603891080574
0.3874551656845996	0.4004569747092908	0.7783193837297541
0.3791449858495778	0.4571229661468218	0.8190125233493765
0.4337696310264427	0.5515351945825803	0.5331393864235144
0.4965011257510886	0.5878182802798632	0.5329448322408887
0.4884974297995682	0.6471303590150210	0.5745216364682613
0.4635224879043444	0.3415910167665811	0.7096651951582725
0.4508292675733870	0.3638110816872341	0.7783682307636759
0.4344781995626666	0.4797499862908097	0.8607343844911249
0.3205335098396077	0.5159960000000012	0.7261184159090945
0.3457315747783255	0.5159960000000012	0.7925650368095916
0.6719577848206771	0.5159960000000012	0.6673762766227986

0.6469634308447623 0.5159960000000012 0.6009211546939902
0.4961803844249181 0.5159960000000012 0.4465672852762978
0.4963453518901495 0.5159960000000012 0.9465591295910251

Atomic coordination of [Cr&(B@C_{60})$_2$_η^5]$_\infty$

[Cr&(B@C_{60})$_2$_η^5]$_\infty$
1.00000000000000
20.0000000000000000 0.0000000000000000 0.0000000000000000
0.0000000000000000 21.2799999999999976 0.0000000000000000
0.0000000000000000 0.0000000000000000 20.0000000000000000
B C Cr
2 120 2
Selective dynamics
Direct
0.5039501595467897 0.2503932469126795 0.4982950383572274
0.5038302171935812 0.7504310149003430 0.4991508546976959
0.6089779827661843 0.1642366909572815 0.3935664828858734
0.6526124142038014 0.1647034311482756 0.5026754078282991
0.6218049784800286 0.2782034154540725 0.3747014289577635
0.5492555725108518 0.1641644154914203 0.3561165413229475
0.5401830117280184 0.0945815188274253 0.5482164975751614
0.5789704324522307 0.1285630576736949 0.5946802277121890
0.6382329095086520 0.2227399070652326 0.6086252495735850
0.6575889831361578 0.2786590245678084 0.5773615670059726
0.4920024314764646 0.1286008419740270 0.3786850982618262
0.4319783136739802 0.1642757449766492 0.3642634926551907
0.4520181961818749 0.2221422236491222 0.3320070702753953
0.5456214107692716 0.1645780153820908 0.6461033328428486
0.5823708286483581 0.2226267396281614 0.6551927767790532
0.6221396157142891 0.3365913518335261 0.5911832836916914
0.3863734434687914 0.3719722810845382 0.5454734483509540
0.3545721362265937 0.2783822207162103 0.5975594502610518
0.3645597781840522 0.3360284273118950 0.4295794911203786

0.4423472326026854	0.4059962062335387	0.5228096822306583
0.4754976190629421	0.2786307581601336	0.6733383779071503
0.4400235204501826	0.2226347389673649	0.6644928037718215
0.3756226179976068	0.1644897967648262	0.5881729477710846
0.3493566152883290	0.1641879206334238	0.5228842399615203
0.5040320241307298	0.4060398098916522	0.5614827009052448
0.5600198268268600	0.4064173551507224	0.5149769427414199
0.5325951686261020	0.4057115468234186	0.4475761774686333
0.4753424019606384	0.1646659565809543	0.6508162284245813
0.4354319619131956	0.1290602736003663	0.6038752688327164
0.3813072987878030	0.1281424666287024	0.4706090814475559
0.3911546247531206	0.3364650415363925	0.6068966148620162
0.3475286683601339	0.3359904269364975	0.4977756970966709
0.3783063314073658	0.2225249690118716	0.6257667352754837
0.4508679617512570	0.3365521186915217	0.6443412160460582
0.4599627226210501	0.4061353858297991	0.4522149255618047
0.4211702943791238	0.3721526084436774	0.4057697172143548
0.3618973931394152	0.2779760468122277	0.3918249006637140
0.3425343154637090	0.2220692201734878	0.4230900879597854
0.5081262071563089	0.3721197211942737	0.6217655257510942
0.5681525704073138	0.3364425167327028	0.6361866487006721
0.5481101874294367	0.2785710809663513	0.6684430043435937
0.4545156218367837	0.3361486503804513	0.3543315854993168
0.4177626066390658	0.2780910972716697	0.3452552154140776
0.3780013346567231	0.1641410771611973	0.4092712003821433
0.6137819021944821	0.1287158716903747	0.4549781124750893
0.6455303368050520	0.2223291286245015	0.4029075877525656
0.6355824884760252	0.1646772350114318	0.5708766585718621
0.5578031295316209	0.0946877967111775	0.4776353355160196
0.5246288493806901	0.2220892853782317	0.3271344605282351
0.5601001601989850	0.2780982484890870	0.3359656270123708
0.6245158110465781	0.3362564160167949	0.4122637312856980
0.6507727819140995	0.3365464395265504	0.4775651149487602

0.4961103451543920	0.0946711965744384	0.4389616869014929
0.4401430716109048	0.0943308669163869	0.4854686843174818
0.4675628235296116	0.0950466921985225	0.5528483469362654
0.5247993083873225	0.3360868197534827	0.3496032396454751
0.5647271314252720	0.3717110599225251	0.3965290375907954
0.6188425869519422	0.3726031608901935	0.5298467724435056
0.6726970594461720	0.2226313894504486	0.4704292695078606
0.6752594844012522	0.2786508176093108	0.5068837198821399
0.3274129384521843	0.2780787884587283	0.5300314782175589
0.3248462164653034	0.2220776609202630	0.4935673810440875
0.6089026916761924	0.6642510342549245	0.3935389943464926
0.6526064242630413	0.6647102263286371	0.5026624786088263
0.6218132026505573	0.7781616893735686	0.3746703300511456
0.5493116932131699	0.6641576378468983	0.3561436392574323
0.5401923174516911	0.5946131812426713	0.5482408565740328
0.5789793294443510	0.6285830943330950	0.5946401548110886
0.6382232303681336	0.7227083690647264	0.6086376356605641
0.6575522817546257	0.7786474683789208	0.5773355091901350
0.4919698256491927	0.6285871342208650	0.3786424699913269
0.4320132609964489	0.6642486864104526	0.3642784158876751
0.4520447314752047	0.7221915661764767	0.3320430457584535
0.5455939062515824	0.6646004475796160	0.6461012775767756
0.5824026705456761	0.7226548057829376	0.6551740895669694
0.6221472861506571	0.8365767097708955	0.5911935775229082
0.3863506443885072	0.8719829960727948	0.5454380712214048
0.3545575622127971	0.7783286991301921	0.5975409170839256
0.3645574445066943	0.8360102230212263	0.4295521265989067
0.4423439581727422	0.9060108615460224	0.5228369378900553
0.4755074055684148	0.7785995424129837	0.6733621733671739
0.4399801722888839	0.7226737847830762	0.6645114235625725
0.3756176156328336	0.6644565473057611	0.5881821667063108
0.3493634794313465	0.6642090992940338	0.5229189842627379
0.5040096460417658	0.9060599365709763	0.5614984487915465

0.5600460483620254	0.9064207875330013	0.5149783613479642
0.5325986352499258	0.9057040448591634	0.4475660342054582
0.4753687616576273	0.6646642555893435	0.6507948813973314
0.4353858534452826	0.6290453492889725	0.6038974004015286
0.3813505554957928	0.6281385964058899	0.4706147443392439
0.3912129065352445	0.8364700145107165	0.6069233909055994
0.3475379393343658	0.8359945761093782	0.4977891951845024
0.3782939967072565	0.7225546965806160	0.6258169934753254
0.4508249944189017	0.8365762864528535	0.6443393967279151
0.4599619246312940	0.9061110306284874	0.4522019492896874
0.4211659119178534	0.8721296805670563	0.4058062471333911
0.3619284859398491	0.7780128508532513	0.3918212417252391
0.3425827109424446	0.7220842022518639	0.4231167918397105
0.5081692398288666	0.8721554525278066	0.6218246815734644
0.5681346977561575	0.8364902347249554	0.6362099891482559
0.5481039777042511	0.7785486746943561	0.6684461885834665
0.4545592006151153	0.8361183336030464	0.3543687423471625
0.4177553744339306	0.7780599179324666	0.3452989397771802
0.3780218809721490	0.6641741890844415	0.4092749698656491
0.6138063307318761	0.6287151295362891	0.4550110598413966
0.6455369664783887	0.7224047381090983	0.4029352040982701
0.6355962641779841	0.6647075897613632	0.5708957739822011
0.5578173144399159	0.5946859742363131	0.4775985686486024
0.5246244152398917	0.7221403508131417	0.3271445839687104
0.5601389203509227	0.7780463484185346	0.3359814251204867
0.6245077124125091	0.8362768598692369	0.4122671910302743
0.6507430282967660	0.8365171663668532	0.4775307715692420
0.4961406404560265	0.5946766119979047	0.4389442853614085
0.4401066090788959	0.5943543151802969	0.4854649032821570
0.4675548515817596	0.5950465973487553	0.5528730720398372
0.5247617931674070	0.8360718712709101	0.3496606109190097
0.5647664421296668	0.8717040155241308	0.3965325893808752
0.6188155627808527	0.8726069182402439	0.5298443629796522

0.6727007978372938	0.7226498741787423	0.4704134201273079
0.6752317137132948	0.7786058498633537	0.5068724485136968
0.3274085930979425	0.7780784841474427	0.5300463542735726
0.3248702959424392	0.7221069023739588	0.4935730256164490
0.5001964067427238	0.0003604854682209	0.5000866912414780
0.5002050963708271	0.5003587679216396	0.5000792834833661
